# The Morphology, Taxonomy, and Phylogenetic Analyses of Five Freshwater Colonial Peritrich Ciliates (Alveolata, Ciliophora), Including the Descriptions of Two New Species

**DOI:** 10.3389/fmicb.2021.718821

**Published:** 2021-08-16

**Authors:** Tong Wu, Zhe Wang, Lili Duan, Hamed El-Serehy, Saleh A. Al-Farraj, Alan Warren, Yujie Liu, Chundi Wang, Borong Lu

**Affiliations:** ^1^Institute of Evolution and Marine Biodiversity, Ocean University of China, Qingdao, China; ^2^College of Fisheries, Ocean University of China, Qingdao, China; ^3^Zoology Department, College of Science, King Saud University, Riyadh, Saudi Arabia; ^4^Department of Life Sciences, Natural History Museum, London, United Kingdom; ^5^Marine College, Shandong University, Weihai, China

**Keywords:** ciliate, sessilid peritrichs, SSU rDNA, systematics, taxonomy

## Abstract

The morphology and phylogeny of two new sessilid species, *Zoothamnium weishanicum* n. sp. and *Epicarchesium sinense* n. sp., two insufficiently known species, *Zoothamnium arbuscula* Ehrenberg, 1831 and *Zoothamnium hentscheli* Kahl, 1935, and a well-known species, *Carchesium polypinum* (Linnaeus, 1767) Ehrenberg, 1838, collected from freshwater habitats of China, were investigated. *Zoothamnium weishanicum* n. sp. is characterized by its inverted bell-shaped zooids, double-layered peristomial lip, alternately branched stalk, and two different-length rows in infundibular polykinety 3 (P3). *Epicarchesium sinense* n. sp. is recognized by its asymmetric-pyriform zooids, single-layered peristomial lip, conspicuous cortical blisters on the pellicle, dichotomously branched stalk, and P3 containing one short inner row and two long outer rows. Based on previous and newly obtained data of the three known species, improved diagnoses and redescriptions are provided including, for the first time, data on the infraciliature of *Z. arbuscula* and *Z. hentscheli*. In addition, we analyzed the phylogeny of each species based on SSU rDNA sequence data.

## Introduction

Ciliated protists (ciliates) are a group of unicellular eukaryotes with high species diversity and a cosmopolitan distribution (Song et al., 2009; Hu et al., 2019). They have been used widely in a variety of fields of investigation including cytology, evolutionary biology, and ecology (Chen et al., 2020; Wang Y.R. et al., 2019, 2020; Zhang et al., 2020; Zhu et al., 2020). Peritrichia Stein, 1859 is probably the most speciose subclass in the class Oligohymenophorea de Puytorac et al., 1974 with more than 1,000 nominal species collected from a wide range of habitats (Kent, 1880–1882; Entz, 1884; Penard, 1922; Kahl, 1935; Foissner et al., 1992; Lu et al., 2019; Wang Z. et al., 2020).

In the classification of Lynn (2008), Peritrichia is composed of two orders: Sessilida Kahl, 1933 and Mobilida Kahl, 1933. The species of order Sessilida are either solitary or colonial and are commonly attached to a substrate *via* a stalk, a scopula, or a lorica (Lynn, 2008). Although investigations of sessilids have been carried out for more than 300 years, many species are poorly described since they are known only from *in vivo* observations and information on their infraciliature, silverline system, and molecular phylogeny is lacking (Kahl, 1935; Precht, 1935; Nenninger, 1948; Sommer, 1951; Stiller, 1971; Bernerth, 1982; Foissner et al., 1992). These insufficient descriptions make the species identification of many sessilids extremely difficult, thus highlighting the need for their reinvestigation based on modern methods (Warren et al., 2018). Furthermore, new species are continuously being reported, suggesting that there is a large undiscovered diversity of sessilids (Canals and Salvadó, 2016; Kühner et al., 2016; Wang et al., 2017; Zhou et al., 2019a,b; Lu et al., 2020; Wu et al., 2020, 2021).

In the present study, five species representing three genera (*Zoothamnium* Bory de St. Vincent, 1824, *Epicarchesium* Jankowski, 1985, and *Carchesium* Ehrenberg, 1831) and two families (Zoothamniidae Sommer, 1951 and Vorticellidae Ehrenberg, 1838) are investigated. *Zoothamnium* is characterized by its colonial habit, transverse silverline system, continuous spasmoneme, and the contraction of the stalk in a “zig-zag” fashion (Bory de St. Vincent, 1824; Corliss, 1979). It contains more than 140 nominal species, about two-thirds of which lack data on their silverline system and/or infraciliature (Ji et al., 2015; Schuster and Bright, 2016; Shen et al., 2017; Lu et al., 2020; Mayen-Estrada and Dias, 2021). *Epicarchesium* is characterized by its colonial habit, discontinuous spasmoneme, tuberculate pellicle, reticulate silverline system, and the contraction of the stalk in a spiral fashion (Jankowski, 1985; Leitner and Foissner, 1997). *Carchesium* is similar to *Epicarchesium* but has a transverse silverline system, and its pellicle is not tuberculate (Ehrenberg, 1831; Kahl, 1935; Shen and Gu, 2016). Compared with *Zoothamnium*, *Epicarchesium* and *Carchesium* are poorly studied in terms of their morphology and phylogenetics and, with the exception of one or two species, morphological information based on modern standards and accurately identified SSU rDNA sequences are lacking for both genera.

During faunal surveys of freshwater ciliates in two widely separated locations in Shandong Province, China, five colonial sessilid peritrichs representing these three genera were isolated, giving the opportunity to investigate them using modern methods. Here we provide detailed morphological information based on the observations of specimens *in vivo* and after silver staining. We also sequenced their small subunit ribosomal DNA (SSU rDNA) and analyzed their phylogenetic relationships.

## Materials and Methods

### Sample Collection

**FIGURE 1:**
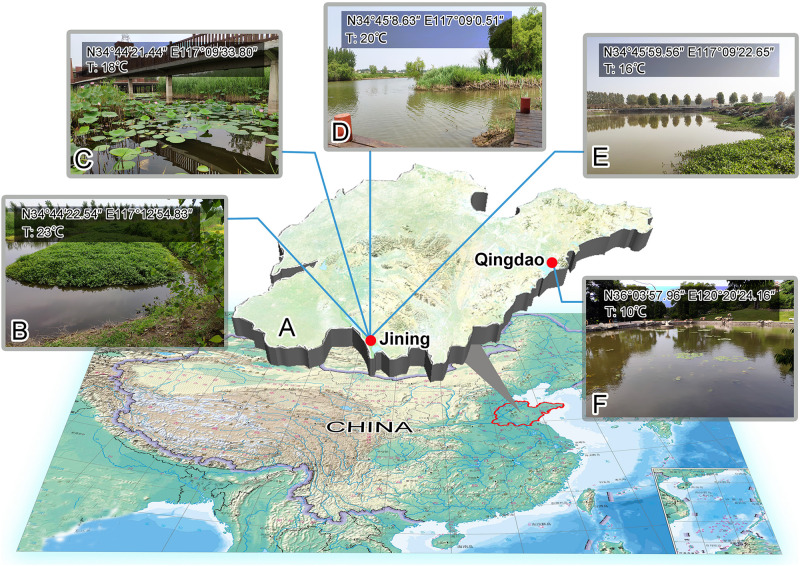
Sampling sites. **(A)** Map of Shandong Province to indicate the locations of the sampling sites. **(B)** Sampling site of *Zoothamnium weishanicum* n. sp., Jining. **(C)** Sampling site of *Zoothamnium arbuscula*, Jining. **(D)** Sampling site of *Zoothamnium hentscheli*, Jining. **(E)** Sampling site of *Epicarchesium sinense* n. sp., Jining. **(F)** Sampling site of *Carchesium polypinum*, Qingdao. T, temperature.

All the species were isolated in 2019 from freshwater habitats in either Weishan or Qingdao, Shandong Province, China (Figure 1A), using glass microscope slides as artificial substrates. Briefly, the slides were fixed onto a frame that was immersed in water at a depth of 1–2 m for 7–10 days to allow colonization by ciliates (Small, 1973).

*Zoothamnium weishanicum* n. sp. was collected on June14, 2019 from Jiangjiaji River in Weishan (34°45′22.54″ N, 117°12′54.83″ E), where the water temperature was 23°C (Figures 1A,B). *Zoothamnium arbuscula* was collected on May 9, 2019 from an outflow of a wetland in Weishan (34°44′21.44″ N, 117°09′33.80″ E), where the water temperature was 18°C (Figures 1A,C). *Zoothamnium hentscheli* was collected on May 18, 2019 from a wharf in Weishan (34°45′8.63″ N; 117°09′0.51″ N), where the water temperature was 20°C (Figures 1A,D). *Epicarchesium sinense* n. sp. was collected on October 23, 2019 from an aquaculture pond in Weishan (34°45′59.56″ N, 117°09′22.65″ E), where the water temperature was 16°C (Figures 1A,E). *Carchesium polypinum* was collected on January 19, 2019 from a freshwater pond in Qingdao (36°03′57.96″ N, 120°20′24.16″ E), where the water temperature was 10°C (Figures 1A,F).

### Investigation of Morphology

Colonies were removed from the slides using acupuncture needles and transferred with glass micropipettes. Live specimens were observed using differential interference contrast microscopy at magnifications of ×40 to ×1,000. The infraciliature was revealed by the protargol staining method (Wilbert, 1975; Ji and Wang, 2018). The silverline system was demonstrated using the “dry” silver nitrate method (Song and Wilbert, 1995; Foissner, 2014). Counts and measurements were performed at ×400–1,000 magnifications. Drawings of live organisms were performed based on actual observations and photomicrographs, while those of stained specimens were made with the help of a drawing device. The terminology is according to Warren (1986) and Foissner et al. (1992).

### DNA Extraction, PCR Amplification, and Sequencing

For each species, five zooids were isolated and washed five times with distilled water to remove potential contamination. Genomic DNA was extracted using DNeasy Blood and Tissue Kit (Qiagen, Hilden, Germany) following the instruction of the manufacturer. The SSU rDNA was amplified using the primers 82F (5′-GAA ACT GCG AAT GGC TC -3′) (Jerome et al., 1996) and 18SR (5′-TGA TCC TTC TGC AGG TTC ACC TAC-3′) (Medlin et al., 1988). Q5^®^ Hot Start High-Fidelity DNA Polymerase (NEB, Ipswich, MA) was used to minimize the possibility of PCR amplification errors. The PCR programs were designed according to Bai et al. (2020). The PCR products were sequenced bidirectionally by Tsingke Biological Technology Company (Qingdao, China).

### Phylogenetic Analyses

The five newly obtained SSU rDNA sequences and 54 sequences of other peritrichs downloaded from GenBank (accession numbers are shown in Figure 12) were used for phylogenetic analyses. Four hymenostomatians (*Glaucoma chattoni* X56533, *Ichthyophthirius multifiliis* U17354, *Tetrahymena corlissi* U17356, and *Tetrahymena pyriformis* EF070254) were selected as outgroup taxa. All the SSU rDNA sequences were aligned using the GUIDANCE2 algorithm^1^ with default parameters (Landan and Graur, 2008; Sela et al., 2015). The two ends of the resulting alignment were trimmed manually in BioEdit v.7.0 (Hall, 1999). The final length of the alignment was 2,287 bp.

Maximum likelihood (ML) analysis with 1,000 bootstrap replicates was computed at CIPRES Science Gateway,^2^ using RAxML-HPC2 on XSEDE v.8.2.10 (Stamatakis, 2014) with GTRGAMMA + I model. Bayesian inference (BI) analysis was carried out using MrBayes v.3.2.6 on XSEDE (Ronquist et al., 2012) on CIPRES Science Gateway with GTR + I + G model selected by JModeltest v.2 (Darriba et al., 2012) under Akaike Information Criterion. Markov chain Monte Carlo simulations were run for 1,000,000 generations with a sample frequency of 100 generations. The first 25% of trees were discarded as burn-in. The run would finish after 1,000,000 generations if the split frequencies were below 0.01. All the remaining trees were used to calculate posterior probabilities using a 50% majority rule consensus. Tree topologies were visualized using MEGA v.7.0 (Kumar et al., 2016). The classification is mainly according to Lynn (2008) and Gao et al. (2016).

## Results

### ZooBank Registration

Present work: urn:lsid:zoobank.org:pub:EC4C6372-5044-40C0-BE0C-790AF40632F0

Subclass Peritrichia Stein, 1859

Order Sessilida Kahl, 1933

Family Zoothamniidae Sommer, 1951

Genus *Zoothamnium* Bory de st. Vincent, 1824

**FIGURE 2:**
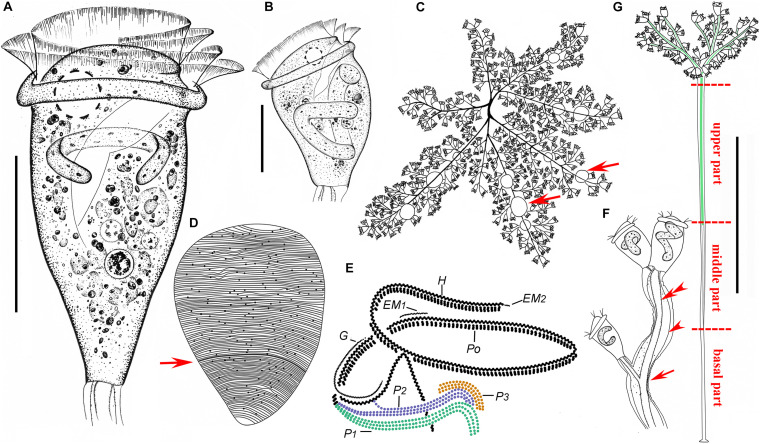
*Zoothamnium arbuscula in vivo***(A–C,F,G)**, after “dry” silver nitrate staining **(D)**, and after protargol staining **(E)**. **(A,B)** Different zooids, showing the shape variation. **(C)** Apical view of a mature colony; arrows mark the macrozooids. **(D)** Silverline system; arrow marks the trochal band. **(E)** Oral ciliature. **(F)** Branch structure; arrow marks the folds on the surface of the stalk, arrowhead marks the sheath of the spasmoneme, and double arrowhead marks the spasmoneme. **(G)** A developing colony; the structure in green is the spasmoneme. EM1, 2, epistomial membrane 1, 2; G, germinal kinety; H, haplokinety; Po, polykinety; P1–3, infundibular polykineties 1–3. Bars: 35 μm in panels **(A,B)** and 900 μm in panel **(G)**.

**FIGURE 3:**
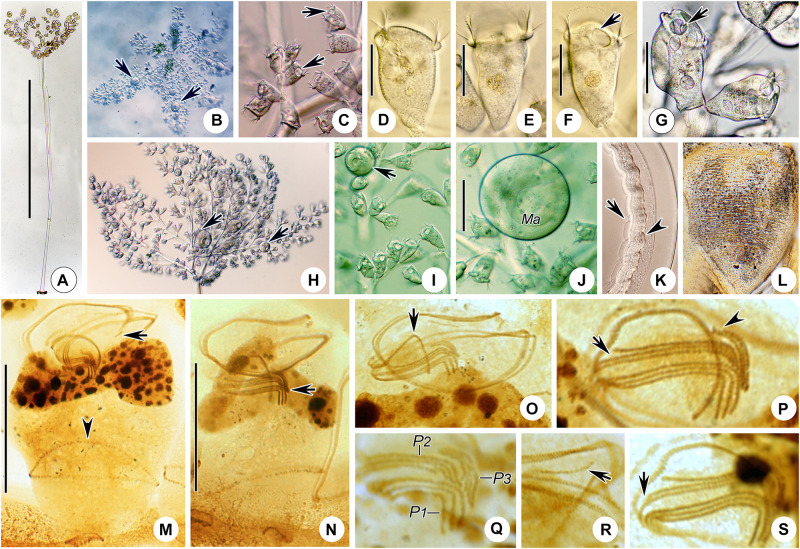
Photomicrographs of *Zoothamnium arbuscula in vivo* **(A–K)**, after “dry” silver nitrate staining **(L)**, and after protargol staining **(M–S)**. **(A)** A developing colony. **(B)** Apical view of a mature colony; arrows mark the macrozooids. **(C–I)** Different zooids showing the shape variation; arrows in panels **(C,F,G)** mark the contractile vacuole, and arrows in panels **(H,I)** mark the macrozooids. **(J)** Macrozooid showing the macronucleus. **(K)** Detail of the stalk; arrow marks the folds on the surface, and arrowhead marks the spasmoneme. **(L)** Silverline system. **(M,N)** Two protargol-stained zooids showing the infraciliature and macronucleus; arrow in panel **(M)** marks epistomial membrane 2, arrowhead in panel **(M)** marks the trochal band, and arrow in panel **(N)** marks the infundibular polykineties. **(O)** Oral ciliature; arrow marks the germinal kinety. **(P,Q)** Infundibular polykineties 1–3 (P1–3); arrow in panel **(P)** marks the abstomal end of P2, and arrowhead in panel **(P)** marks the abstomal end of P3. **(R,S)** Part of the oral ciliature; arrow in panel **(R)** marks epistomial membrane 1, and arrow in panel **(S)** marks the abstomal end of P2. Bars: 900 μm in panel **(A)**, 35 μm in panels **(D–G)**, 100 μm in panel **(J)**, and 25 μm in panels **(M,N)**.

*Zoothamnium arbuscula* Ehrenberg, 1831

(Figures 2A–G, 3A–S and Table 1)

**TABLE 1:** Morphometrical characterization of five colonial sessilid peritrich species based on specimens *in vivo* (except where stated).

Character	Species	Max	Min	Mean	SD	CV	*n*
Zooid length (μm)	*Z. weishanicum* n. sp.	90	55	76.0	11.01	14.5	10
	*Z. arbuscula*	80	50	62.3	9.27	14.9	14
	*Z. hentscheli*	80	50	65.4	10.30	15.7	13
	*E. sinense* n. sp.	60	45	52.0	4.22	8.1	10
	*C. polypinum*	65	35	51.2	7.68	15.0	13
Zooid width (μm)	*Z. weishanicum* n. sp.	45	30	37.5	5.40	14.4	10
	*Z. arbuscula*	65	30	40.4	11.45	28.3	14
	*Z. hentscheli*	40	30	32.7	3.88	11.9	13
	*E. sinense* n. sp.	40	30	33.0	4.22	12.8	10
	*C. polypinum*	60	35	49.2	7.03	14.3	13
Diameter of peristomial lip (μm)	*Z. weishanicum* n. sp.	50	30	38.0	7.89	20.8	10
	*Z. arbuscula*	55	35	41.9	5.96	14.2	14
	*Z. hentscheli*	45	35	40.0	2.89	7.2	13
	*E. sinense* n. sp.	40	35	38.5	2.41	6.3	10
	*C. polypinum*	85	60	74.6	6.91	9.3	13
Height of colony (μm)	*Z. weishanicum* n. sp.	1,400	1,000	1,275.0	184.84	14.5	4
	*Z. arbuscula*	3,500	2,800	3,233.3	378.59	11.7	3
	*Z. hentscheli*	1,500	750	1,125.0	530.33	47.1	2
	*E. sinense* n. sp.	750	400	562.5	165.2	29.4	4
	*C. polypinum*	2,500	2,400	2,450.0	70.71	2.9	2
Number of silverlines, peristome to trochal band^a^	*Z. weishanicum* n. sp.	55	51	53.3	2.08	3.9	3
	*Z. arbuscula* ^b^	83	72	76.3	5.86	7.7	3
	*Z. hentscheli*	65	65	65.0	–	–	1
	*E. sinense* n. sp.^b^	37	37	37.0	–	–	1
	*C. polypinum*	77	71	74.0	3.00	4.1	3
Number of silverlines, trochal band to scopula^a^	*Z. weishanicum* n. sp.	33	32	32.7	0.58	1.8	3
	*Z. arbuscula* ^b^	52	46	49.7	3.21	6.5	3
	*Z. hentscheli*	30	30	30	–	–	1
	*E. sinense* n. sp.^b^	23	23	23.0	–	–	1
	*C. polypinum*	47	36	42.0	5.57	13.3	3

*CV, coefficient of variation in %; Max, maximum; Min, minimum; Mean, arithmetic mean; *n*, number of specimens investigated; SD, standard deviation; –, data not available.*

*^*a*^Rough data.*

*^*b*^Data based on “dry” silver nitrate-stained specimens. All data are based on the populations investigated in the present study.*

1831 *Zoocladium arbuscula* n. sp.—Ehrenberg, Abh. dt. Akad. Wiss. Berl., Jahr 1831: 94 (original description)

1838 *Zoothamnium arbuscula* Ehrenberg, 1831—Ehrenberg, Infusionsthierchen, p. 289 (revision)

1892 *Zoothamnium arbuscula* Ehrenberg, 1831—Entz, Math. Naturw. Ber. Ung., 10: 5 (detailed redescription based on observation *in vivo*)

1903 *Zoothamnium geniculatum* n. sp.—Ayrton, J. Quekett Microsc. Club, 8: 407 (synonym; population in England, with illustrations)

1925 *Zoothamnium geniculatum* Ayrton, 1903—Wesenberg-Lund, K. danske Vidensk. Selsk. Skr., 10: 1 (redescription and life history based on observation *in vivo*)

1929 *Zoothamnium arbuscula* Ehrenberg, 1831—Furssenko, Arch. Protistenk.,67: 377–495

1935 *Zoothamnium arbuscula* Ehrenberg, 1839—Kahl, Tierwelt Dt1., 30: 745, Figures 140 (15–17), 141 (4–8) (revision)

1962 *Zoothamnium arbuscula* Ehrenberg, 1831—Biernacka, Polskie Arch. Hydrobiol., 10: 67, Figure 134 (habitat; population density and biomass)

1980 *Zoothamnium arbuscula* Ehrenberg, 1831—Müller, Mikrokosmos, 69: 222, 334 (redescription based on German population, with photomicrographs)

1988 *Zoothamnium arbuscula* Ehrenberg, 1831—Xu, Chin. J. Zool., 23: 8 (ecological investigation)

1992 *Zoothamnium arbuscula* Ehrenberg, 1831—Foissner et al., Informationsberichte des Bayer. Landesamtes für Wasserwirtschaft, 5/92: 158–162, Figures 1–22 (revision)

1996 *Zoothamnium arbuscula* Ehrenberg, 1831—Foissner and Berger, Freshw. Biol., 35: 385 (illustrations)

2016 *Zoothamnium arbuscula* Ehrenberg, 1831—Shen and Gu, Fauna Sinica: Invertebrata 45: 187–188, Figure 223 (redescription)

Although *Zoothamnium arbuscula* has been reported many times, details of its infraciliature were hitherto unknown (Ehrenberg, 1831, 1838; Ayrton, 1903; Wesenberg-Lund, 1925; Furssenko, 1929; Kahl, 1935; Biernacka, 1962; Müller, 1980; Xu, 1988; Foissner et al., 1992; Foissner and Berger, 1996; Shen and Gu, 2016). Here we provide details of its infraciliature and an improved diagnosis based on previous reports and our new data.

#### Improved Diagnosis

The colony was up to 3,500 μm high. Accessory branches radiate from the main stalk, forming an inverted dome-like outline, with micro- and macrozooids. The microzooids were inverted bell-shaped, 40–80 × 30–65 μm *in vivo*. The macrozooids were nearly globular, up to 150 μm in diameter. The peristomial lip was single-layered and strongly everted. One contractile vacuole was dorsally located, at the same level as the peristomial lip. The macronucleus is typically C-shaped and transversely oriented. The infundibular polykinety 3 (P3) consists of three equal-length rows, terminating adstomally above infundibular polykinety 1 (P1). Transverse silverlines numbered about 75 from the peristome to the trochal band and about 50 from trochal band to scopula. Freshwater is the habitat.

### Description Based on Weishan Population

The colony was with micro- and macrozooids. The microzooids were inverted bell-shaped, about 50–80 × 30–65 μm *in vivo* (Figures 2A,B, 3C–G,I). The peristomal lip was about 35–40 μm in diameter, single-layered, and strongly everted (Figures 2A,B, 3C–G,I,J). The peristomal disc was moderately elevated in fully extended zooids (Figures 2A, 3D–G). The macrozooids were nearly globular, about 150 μm in diameter (Figures 2C, 3B,H,I,J). The pellicular striations were extremely fine.

The cytoplasm was colorless, usually containing numerous vacuoles with yellow and/or green contents, possibly the remains of ingested algae. A single contractile vacuole was located at the dorsal wall of the infundibulum, about the same level as the peristomial lip (Figures 2A,B, 3F,G). The macronucleus of most microzooids was typically C-shaped and transversely oriented (Figures 2A, 3M,N), the macronucleus of microzooids at the end of the branches varied in shape (Figures 2B,F, 3G), and the macronucleus of macrozooids was usually C-shaped (Figure 3J). A micronucleus was not observed.

The colony was up to 3,500 μm tall, usually containing more than 100 zooids and with accessory branches that radiate from the apical end of the main stalk forming an inverted dome-like outline (Figures 2C,G, 3A,B,H). The main stalk consists of three parts: a basal part without spasmoneme, about 25 μm across; a middle part with a central bundle of transparent fibrils, about 40 μm across; and an upper part with sturdy spasmoneme, about 60 μm in diameter (Figure 2G). The spasmoneme was covered by a sheath with a rough surface, comprising bundles of fibrils (stalk myonemes) within a transparent membrane, which was about 40 μm across its widest point (Figures 2F, 3K).

The oral ciliature was of the usual type for sessilid peritrichs. Haplokinety and polykinety make approximately 1.25 circuits around the peristome before entering the infundibulum where they make a further circuit (Figures 2E, 3M–O). Three infundibular polykineties (P1–P3) were each composed of three rows of kinetosomes (Figures 2E, 3M–Q). P1 is continuous with polykinety and terminates adstomally below P2 and P3, with P2 about twice the length of P3 and terminating adstomally at the convergence of P1 and P3 (Figures 2E, 3M–Q). The rows of P1 were nearly equal in length (Figures 2E, 3O,P). The inner two rows of P2 converge abstomally with P1, and the outer row of P2 separated abstomally from the inner two rows (Figures 2E, 3P,S). P3 consists of three almost-equal-length rows of kinetosomes, terminating adstomally above P1 (Figures 2E, 3N–Q). There were two epistomial membranes (EM1 and EM2): EM1, long, was located at the entrance of the infundibulum (Figures 2E, 3O,R), while EM2 was located in front of the distal ends of haplokinety and polykinety (Figures 2E, 3M). The germinal kinety lies parallel to haplokinety in the upper half of the infundibulum (Figures 2E, 3O). The trochal band consists of dikinetids, located about two-thirds down the length of zooid (Figures 2D, 3M,N).

The silverline system consists of closely spaced transverse silverlines, numbering about 75 (*N* = 3) from the peristome to the trochal band and about 50 (*N* = 3) from the trochal band to the scopula (Figures 2D, 3L).

**FIGURE 4:**
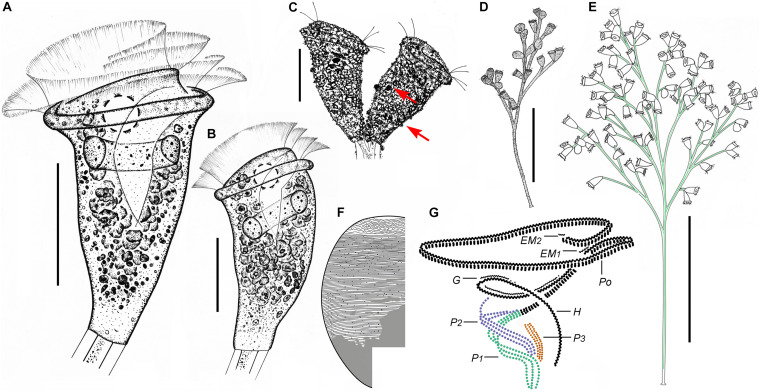
*Zoothamnium hentscheli in vivo***(A–E)**, after “dry” silver nitrate staining **(F)**, and after protargol staining **(G)**. **(A,B)** Different zooids showing the shape variation. **(C)** Arrows mark the pellicle with attached detritus. **(D)** A developing colony covered with detritus. **(E)** A mature colony; the structure in green is the spasmoneme. **(F)** Silverline system. **(G)** Oral ciliature. EM1, 2, epistomial membrane 1, 2; G, germinal kinety; H, haplokinety; Po, polykinety; P1–3, infundibular polykineties 1–3. Bars: 35 μm in panels **(A–C)**, 300 μm in panel **(D)**, and 500 μm in panel **(E)**.

**FIGURE 5:**
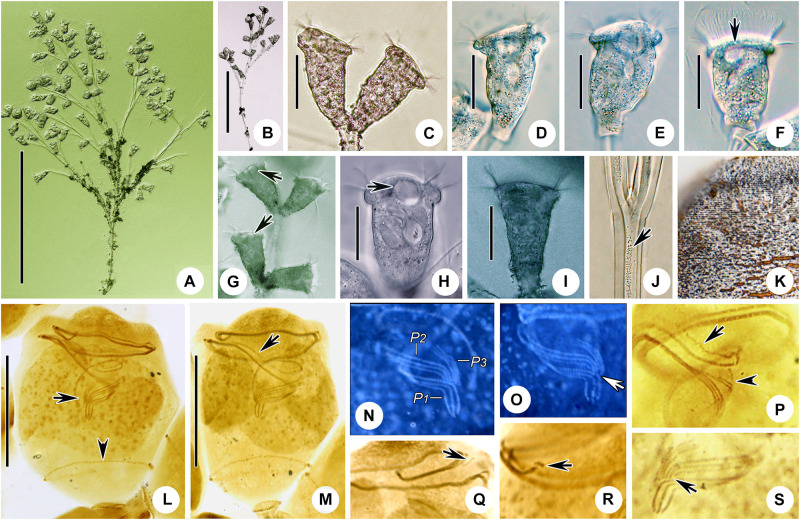
Photomicrographs of *Zoothamnium hentscheli in vivo* **(A–J)**, after “dry” silver nitrate staining **(K)**, and after protargol staining **(L–S)**. **(A)** A mature colony. **(B)** A developing colony. **(C–I)** Different zooids showing the shape variation; arrows mark the contractile vacuole. **(J)** Detail of stalk; arrow marks the spasmoneme. **(K)** Silverline system. **(L,M)** Two protargol-stained zooids showing the ciliature; arrow in panel **(L)** marks the P3, arrowhead in panel **(L)** marks the trochal band, and arrow in panel **(M)** marks the germinal kinety. **(N,O)** Infundibular polykineties 1–3 (P1–3) (image processed by the reverse function *via* Photoshop); arrow marks the adstomal end of P3. **(P–S)** Part of the oral ciliature; arrow in panel **(P)** marks the germinal kinety, arrowhead in panel **(P)** marks the abstomal end of P2, arrow in panel **(Q)** marks epistomial membrane 1, arrow in panel **(R)** marks epistomial membrane 2, and arrow in panel **(S)** marks the adstomal end of P2. Bars: 500 μm in panel **(A)**, 300 μm in panel **(B)**, 35 μm in panels **(C–F,H,I)**, and 25 μm in panels **(L,M)**.

*Zoothamnium hentscheli* Kahl, 1935

(Figures 4A–G, 5A–S and Table 1)

1916 *Zoothamnium* spec. a.—Hentschel, Mitt. Zool. Mus. Hamb., 33: 16–17, Figure 2 (description, unnamed)

1935 *Zoothamnium hentscheli* Kahl, Tierwelt Dtl., 30: 747 [establishment of a new species according to the description of Hentschel (1916); revision]

1952 Zoothamnium *hentscheli* Kahl, 1935—Hammann, Arch. Hydrobiol., 47: 217 (redescription, with illustrations)

1988 Zoothamnium *hentscheli* Ehrenberg, 1831 –Xu, Chin. J. Zool., 23: 8 (ecological investigation)

*Zoothamnium hentscheli* was first described by Hentschel (1916) without a species name (*Zoothamnium* spec. a). Kahl (1935) named it *Zoothamnium hentscheli*. To date, this species has been reported several times; however, the details of its infraciliature remain unknown, which necessitates a reinvestigation (Hentschel, 1916; Kahl, 1935; Hammann, 1952; Xu, 1988). We collected a population of this species from Weishan and made a detailed redescription. An improved diagnosis based on previous and present data is also supplied.

#### Improved Diagnosis

The colony was up to 1,500 μm high. The stalk was alternately branched. The zooids were inverted bell-shaped, about 50–85 × 30–45 μm *in vivo*, and often densely covered with detritus. The peristomial lip was single-layered and moderately everted. The peristomial disc was slightly elevated. A contractile vacuole was dorsally located at the same level as the peristomial lip. The macronucleus was C-shaped and transversely oriented. The infundibular polykinety 3 (P3) consists of three approximately equal-length rows and terminates adstomally above infundibular polykinety 1 (P1). Transverse silverlines numbered about 65 from the peristome to the trochal band and about 30 from the trochal band to the scopula. Freshwater is the habitat.

### Description Based on Weishan Population

The zooids were usually inverted bell-shaped, 50–80 × 30–40 μm *in vivo*, and often densely covered with detritus (Figures 4A–C, 5C–I). The peristomial lip was about 35–45 μm in diameter, single-layered, and moderately everted (Figures 4A,B, 5C–I). The peristomial disc was slightly elevated above the peristomial lip in fully extended zooids (Figures 4A,B, 5C–E,G–I). The pellicular striations were extremely fine (Figures 4F, 5K).

The cytoplasm was colorless and contained several gray or colorless granules. A single contractile vacuole was located at the dorsal wall of the infundibulum at the same level as the peristomial lip (Figures 4A,B, 5F–H). The macronucleus was C-shaped and transversely oriented (Figures 4A,B, 5L,M). The micronucleus not observed.

The colony was up to 2,500 μm tall. The stalk alternately branched. The branches progressively narrowed and shortened from the main stalk to the terminal branches (Figures 4D,E, 5A,B). The spasmoneme was with numerous mitochondria (Figure 5J).

The oral ciliature was of the usual type for sessilid peritrichs. Haplokinety and polykinety make approximately 1.25 circuits around the peristome before entering the infundibulum (Figures 4G, 5L,M). The infundibular polykineties (P1–P3) were of three rows (Figures 4G, 5L–O,S). The rows of P1 were nearly equal in length (Figures 4G, 5L,N,O,S). The adstomal end of P2 terminates at the convergence of P1 and P3 (Figures 4G, 5L–O,S). The abstomal end of the inner row of P2 converges with P1; the abstomal end of the outer row of P2 was detached from the inner two rows (Figures 4G, 5P). P3 terminates adstomally above P1, with the rows equal in length (Figures 4G, 5L–O,S). There were two epistomial membranes (EM1 and EM2): EM1, located at the entrance of the infundibulum (Figures 4G, 5Q), and EM2, located near the distal ends of haplokinety and polykinety (Figures 4G, 5R). Germinal kinety lies parallel to haplokinety in the upper half of the infundibulum (Figures 4G, 5L,M). The trochal band consists of dikinetids, located about two-thirds down the length of zooid (Figures 5L,M).

The silverline system consists of closely spaced transverse silverlines, numbering about 65 (*N* = 1) from the peristome to the trochal band and about 30 (*N* = 1) from the trochal band to the scopula (Figures 4F, 5K).

**FIGURE 6:**
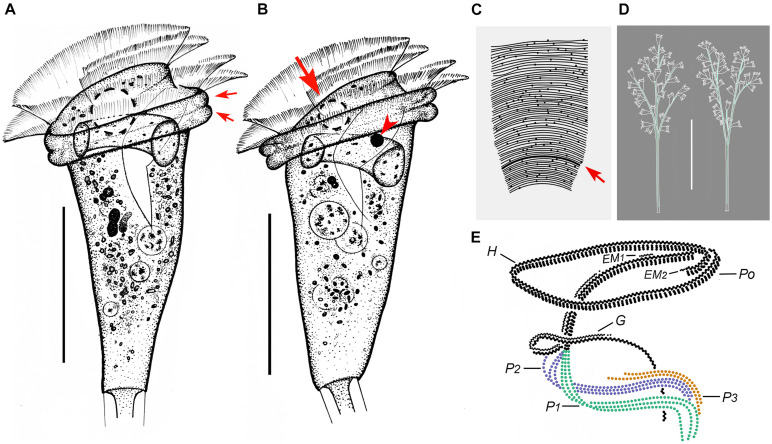
*Zoothamnium weishanicum* n. sp. *in vivo* **(A,B,D)**, after “dry” silver nitrate staining **(C)**, and after protargol staining **(E)**. **(A,B)** Different zooids showing the shape variation; arrows in panel **(A)** mark the double-layered peristomial lip, arrow in panel **(B)** marks the contractile vacuole, and arrowhead in panel **(B)** marks the micronucleus. **(C)** Silverline system; arrow marks the trochal band. **(D)** Two mature colonies. **(E)** Oral ciliature. EM1, 2, epistomial membrane 1, 2; G, germinal kinety; H, haplokinety; Po, polykinety; P1–3, infundibular polykineties 1–3. Bars: 40 μm in panels **(A,B)** and 500 μm in panel **(D)**.

**FIGURE 7:**
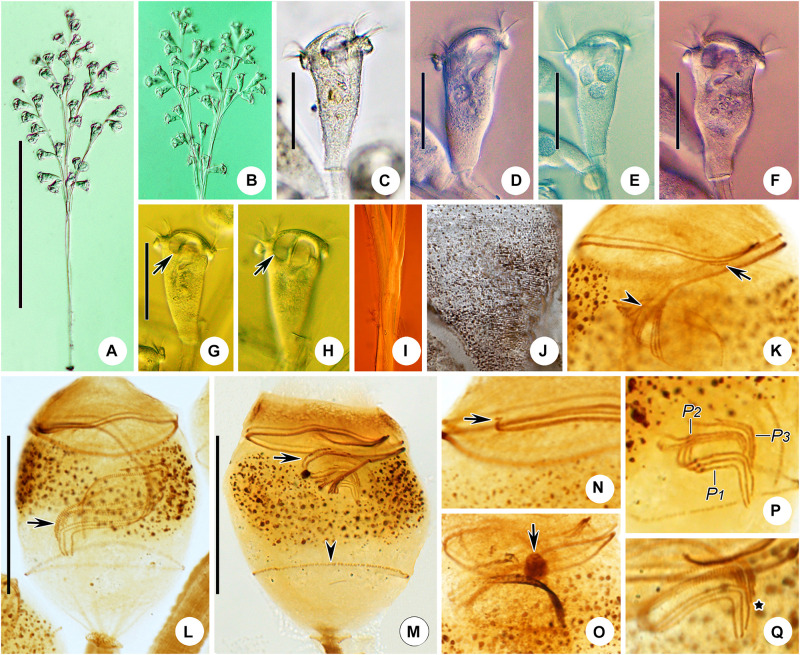
Photomicrographs of *Zoothamnium weishanicum* n. sp. *in vivo* **(A–I)**, after “dry” silver nitrate staining **(J)**, and after protargol staining **(K–Q)**. **(A,B)** Two mature colonies. **(C–H)** Different zooids showing the shape variation; arrows mark the contractile vacuole. **(I)** Detail of stalk. **(J)** Silverline system. **(K)** Part of the oral ciliature; arrow marks epistomial membrane 1, and arrowhead marks the abstomal end of P2. **(L,M)** Two protargol-stained zooids showing the infraciliature; arrow in panel **(L)** marks P3, arrow in panel **(M)** marks the germinal kinety, and arrowhead in panel **(M)** marks the trochal band. **(N)** Part of the oral ciliature; arrow marks epistomial membrane 2. **(O)** Part of protargol-stained zooid; arrow marks the micronucleus. **(P,Q)** Infundibular polykineties 1–3 (P1–3); asterisk marks the adstomal end of P3. Bars: 700 μm in panel **(A)**, 40 μm in panels **(C–G)**, and 30 μm in panels **(L,M)**.

*Zoothamnium weishanicum* n. sp.

(Figures 6A–E, 7A–Q and Table 1)

#### Diagnosis

The colony was up to 1,400 μm high. The stalk was alternately branched. The zooids were inverted bell-shaped, usually 55–90 × 30–45 μm *in vivo*. The peristomial lip was double-layered and strongly everted. The peristomial disc was moderately elevated. A single contractile vacuole was dorsally located, at the same level as the peristomial lip. The macronucleus was C-shaped and transversely oriented. The infundibular polykinety 3 (P3) consists of two different-length rows of kinetosomes and terminates adstomally above infundibular polykinety 1 (P1). There were transverse silverlines numbering about 55 from the peristome to the trochal band and about 33 from the trochal band to the scopula.

#### Type Locality

Jiangjiaji River in Weishan (34°45′22.54″ N, 117°12′54.83″ E), Jining, China (Figures 1A,B).

#### Deposition of Slides

One protargol slide (registration number: WT2019061401–01) with the holotype specimen circled in ink, a second protargol slide with paratype specimens (registration number: WT2019061401–02) and one “dry” silver nitrate slide with paratype specimens (registration number: WT2019061401–03), were deposited in the Laboratory of Protozoology, Ocean University of China (OUC), Qingdao, China.

#### Etymology

The species–group name “*weishanicum*” refers to the area (Weishan) where the sample was collected.

#### Zoobank Registration

*Zoothamnium weishanicum* n. sp.: urn:lsid:zoobank.org:act:CF02 AEF7-8CDC-4F99-84B7-1F8240437B46

### Description

The zooids were inverted bell-shaped, about 55–90 × 30–45 μm *in vivo* (Figures 6A,B, 7C–H). The peristomial lip was about 30–50 μm in diameter, double-layered, and strongly everted (Figures 6A,B, 7C–E). The peristomial disc convex was clearly elevated above the peristomial lip in fully extended zooids (Figures 6A,B, 7C–H). The pellicular striations were extremely fine (Figure 7J).

The cytoplasm was colorless, usually containing numerous vacuoles with yellow and/or green contents, possibly the remains of ingested algae. A contractile vacuole was situated at the dorsal wall of the infundibulum, at the same level as the peristomial lip (Figures 6A,B, 7G,H). The macronucleus was C-shaped and transversely oriented (Figures 6A,B, 7L,M). The micronucleus was located within the curvature of the macronucleus (Figures 6B, 7O).

The colony was up to 1,400 μm tall, usually with fewer than 50 zooids. The stalk was alternately branched; the branches progressively narrowed and shortened from the main stalk to the terminal branches (Figures 6D, 7A). The stalk sheath was colorless, with inconspicuous longitudinal striations (Figure 7I).

The oral ciliature was typical for sessilid peritrichs. Haplokinety and polykinety make approximately 1.5 circuits around the peristome and a further turn within the infundibulum (Figures 6E, 7L,M). P1 and P2 had three rows each; P3 had two rows (Figures 6E, 7L,M,P,Q). The three rows of P1 were nearly equal in length. P2 terminates adstomally at the convergence of P1 and P3 (Figures 6E, 7L,M,P,Q). The abstomal ends of the inner two rows in P2 converge with P1 and diverge from the outer row (Figures 6E, 7K). The inner row of P3 was longer than the outer row which terminates adstomally above the inner row (Figures 6E, 7P,Q). There were two epistomial membranes (EM1 and EM2): EM1 was located at the entrance of the infundibulum (Figures 6E, 7K); EM2 was located close to the distal ends of haplokinety and polykinety (Figures 6E, 7N). The germinal kinety runs parallel to haplokinety in the upper half of the infundibulum (Figures 6E, 7L,M). The trochal band consists of dikinetids, located about two-thirds down the length of zooid (Figures 7L,M).

The silverline system consists of closely spaced transverse silverlines, numbering about 55 (*N* = 3) from the peristome to the trochal band and about 33 (*N* = 3) from the trochal band to the scopula (Figures 6C, 7J).

Family Vorticellidae Ehrenberg, 1838

Genus *Epicarchesium* Jankowski, 1985

**FIGURE 8:**
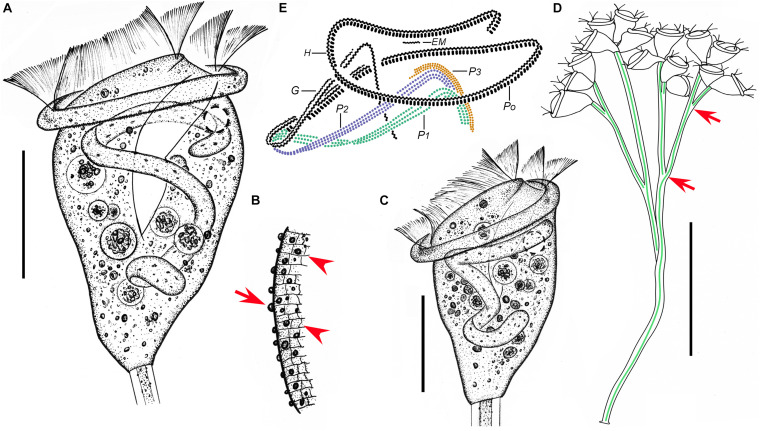
*Epicarchesium sinense* n. sp. *in vivo* **(A–D)** and after protargol staining **(E)**. **(A,C)** Different zooids showing the shape variation. **(B)** Detail of the pellicle; arrow marks a tubercle on the surface, and arrowheads mark the reticulate pattern. **(D)** A mature colony; arrows mark the discontinuous spasmoneme (in green). **(E)** Oral ciliature. EM, epistomial membrane; G, germinal kinety; H, haplokinety; Po, polykinety; P1–3, infundibular polykineties 1–3. Bars: 30 μm in panels **(A,C)** and 250 μm in panel **(D)**.

**FIGURE 9:**
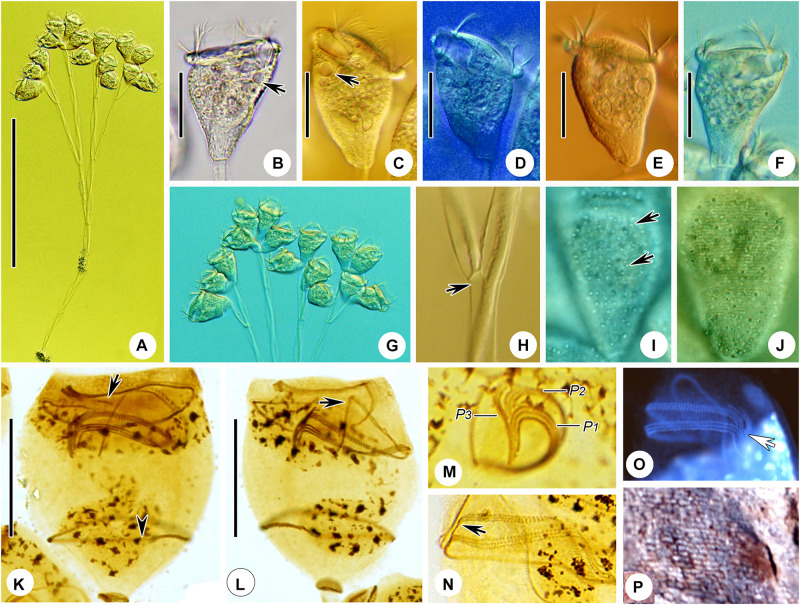
Photomicrographs of *Epicarchesium sinense* n. sp. *in vivo* **(A–J)**, after protargol staining **(K–O)**, and after “dry” silver nitrate staining **(P)**. **(A)** A mature colony. **(B–G)** Different zooids showing the shape variation; arrows mark the contractile vacuole. **(H)** Detail of the stalk; arrow marks the discontinuous spasmoneme. **(I)** Detail of the pellicle; arrows mark the tubercles. **(J)** Reticulate pellicle. **(K,L)** Two protargol-stained zooids showing the infraciliature; arrow in panel **(K)** marks the epistomial membrane, arrowhead in panel **(K)** marks the trochal band, and arrow in panel **(L)** marks the haplokinety. **(M)** Infundibular polykineties 1–3 (P1–3). **(N)** Part of the oral ciliature; arrow marks the abstomal end of P2. **(O)** Part of the oral ciliature (image processed by the reverse function *via* Photoshop); arrow marks P3. **(P)** Silverline system. Bars: 250 μm in panel **(A)**, 30 μm in panels **(B–F)**, and 20 μm in panels **(K,L)**.

*Epicarchesium sinense* n. sp.

(Figures 8A–E, 9A–P and Table 1)

#### Diagnosis

The colony was up to 750 μm tall. The stalk was dichotomously branched. The zooids were asymmetric pyriform, about 45–60 × 30–40 μm *in vivo*. The peristomial lip was single-layered and everted. The peristomial disc was slightly elevated. A contractile vacuole was ventrally located below the level of the peristomial lip. The macronucleus was J-shaped. The infundibular polykinety 3 (P3) has three rows and terminates adstomally at the same level as infundibular polykinety 1 (P1). The inner row of P3 was about half the length of the other two rows. The reticulate silverlines system, with about 37 transverse silverlines from the peristome to the trochal band and about 23 from the trochal band to the scopula. Freshwater is the habitat.

#### Type Locality

A freshwater aquaculture pond in Lake Weishan (34°45′59.56″ N, 117°09′22.65″ E), Jining, China (Figures 1A,E).

#### Deposition of Slides

One protargol slide (registration number: WT2019102301–01) with the holotype specimen circled in ink, a second protargol slide with paratype specimens (registration number: WT2019102301–02), and one “dry” silver nitrate slide with paratype specimens (registration number: WT2019102301–03) were deposited in the Laboratory of Protozoology, Ocean University of China (OUC), Qingdao, China.

#### Etymology

The species–group name “*sinense*” refers to the country (China) where it was first isolated.

#### Zoobank Registration

*Epicarchesium sinense* n. sp.: urn:lsid:zoobank.org:act:3201 DFC4-131A-4444- BB3B-F7822C6CD327

### Description

The zooids were asymmetric pyriform, 45–60 × 30–40 μm *in vivo* (Figures 8A,C, 9B–G). The peristomial lip was about 35–40 μm in diameter, single-layered, and moderately everted (Figures 8A,C, 9B–F). The peristomial disc was slightly elevated above the peristomial lip in fully extended zooids (Figures 8A,C, 9B–F). The pellicle was reticulate, and the tubercles were ca. 0.5–1.5 μm in diameter (Figures 8B, 9I,J).

The cytoplasm was colorless, usually containing numerous vacuoles with yellow and/or green contents, possibly the remains of ingested algae. A single contractile vacuole was located at the ventral wall of the infundibulum below the level of the peristomial lip (Figures 8A,C, 9B,C). The macronucleus was J-shaped (Figures 8A,C, 9K,L). A micronucleus was not observed.

The colony was up to 750 μm tall, usually with fewer than 20 zooids. The stalk was dichotomously branched. The spasmoneme is discontinuous and extends throughout the colony (Figures 8D, 9A).

The oral ciliature was genus-typical. Haplokinety and polykinety make approximately 1.25 circuits around the peristome before entering the infundibulum (Figures 8E, 9K,L). All three infundibular polykineties (P1–P3) were of three rows (Figures 8E, 9M,O). P2 terminates adstomally at the convergence of P1 and P3 (Figures 8E, 9K–M,O). P2 converges abstomally with P1 (Figures 8E, 9N). P3 terminates adstomally at the same level as P1; the inner row of P3 was about half the length of the other two rows, and the inner row terminates abstomally ahead of the other two rows (Figures 8E, 9K,M,O). Only one epistomial membrane was observed, located at the entrance of the infundibulum (Figures 8E, 9K). The germinal kinety lies parallel to haplokinety in the upper half of the infundibulum (Figures 8E, 9K,L). The trochal band consists of dikinetids, located about two-thirds of the way down the length of zooid (Figures 9K,L).

The silverline system consists of reticulate silverlines, with about 37 (*N* = 1) transverse silverlines between the peristome and the trochal band and 23 (*N* = 1) between the trochal band and the scopula (Figure 9P).

Genus *Carchesium* Ehrenberg, 1831

**FIGURE 10:**
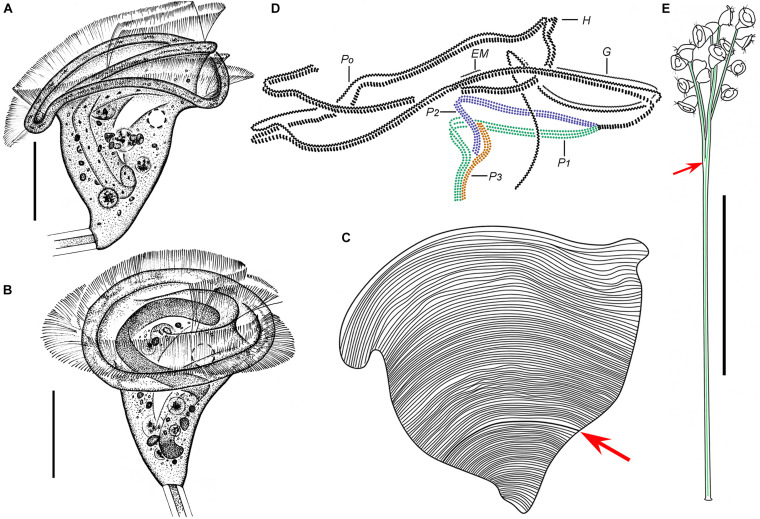
*Carchesium polypinum in vivo***(A–C,E)** and after protargol staining **(D)**. **(A,B)** Different zooids showing the shape variation. **(C)** Pellicular striations; arrow marks the trochal band. **(D)** Oral ciliature. **(E)** A developing colony; arrows mark the discontinuous spasmoneme (in green). EM, epistomial membrane; G, germinal kinety; H, haplokinety; Po, polykinety; P1–3, infundibular polykineties 1–3. Bars: 25 μm in panels **(A,B)** and 700 μm in panel **(E)**.

**FIGURE 11:**
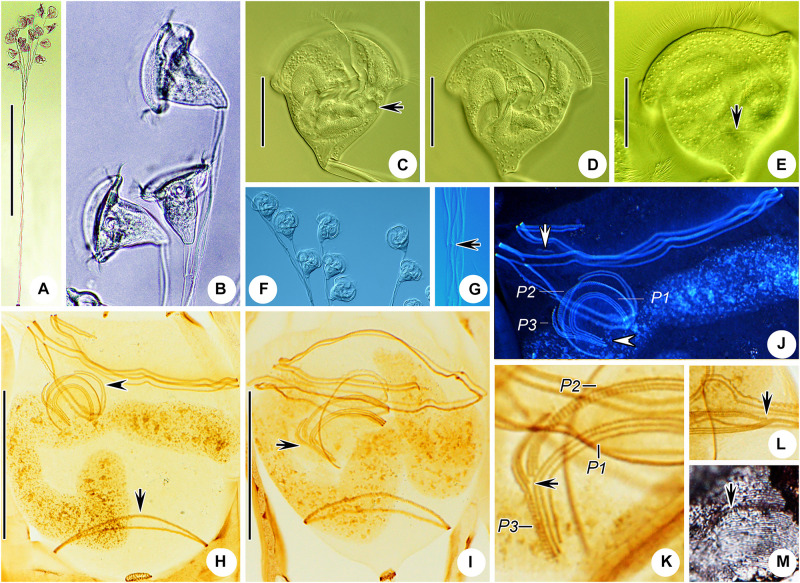
Photomicrographs of *Carchesium polypinum in vivo* **(A–G)**, after protargol staining **(H–L)**, and after “dry” silver nitrate staining **(M)**. **(A)** A developing colony. **(B–D,F)** Different zooids showing the shape variation; arrow marks the contractile vacuole. **(E)** Pellicular striations; arrow marks the trochal band. **(G)** Detail of the stalk; arrow marks the discontinuous spasmoneme. **(H,I)** Two protargol-stained zooids showing the infraciliature; arrow in panel **(H)** marks the trochal band, arrowhead in panel **(H)** marks the germinal kinety, and arrow in panel **(I)** marks the infundibular polykinety 3. **(J)** Oral ciliature; arrow marks the epistomial membrane, and arrowhead marks the infundibular polykineties (image processed by the reverse function *via* Photoshop). **(K)** Infundibular polykineties 1–3 (P1–3); arrow marks the adstomal end of the inner row of P3. **(L)** Part of the oral ciliature; arrow marks the abstomal end of P2. **(M)** Silverline system; arrow marks the trochal band. Bars: 800 μm in panel **(A)**, 30 μm in panels **(C–E)**, and 30 μm in panels **(H,I)**.

*Carchesium polypinum* (Linnaeus, 1758) Ehrenberg, 1830

(Figures 10A–E, 11A–M and Table 1)

**FIGURE 12:**
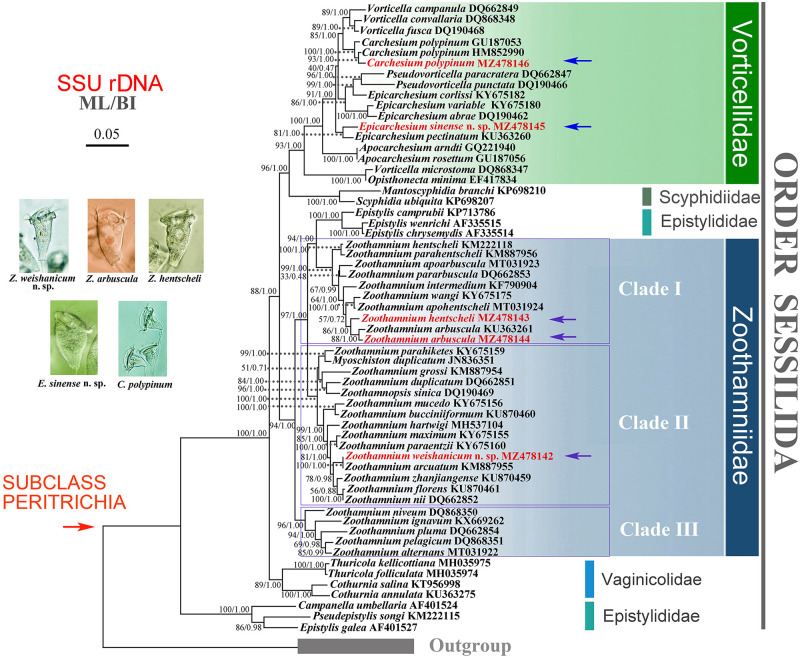
Maximum likelihood (ML) tree inferred from SSU rDNA sequences, revealing the phylogenetic positions of *Zoothamnium weishanicum* n. sp., *Z*. *arbuscula*, Z. *hentscheli*, *Epicarchesium sinense* n. sp., and *Carchesium polypinum* (marked by arrows and red font). Numbers near the nodes denote maximum bootstrap values of ML out of 1,000 replicates and posterior probability of Bayesian inference. The scale bar indicates five substitutions per 100 nucleotide positions.

1758 *Sertularia polypina* Linnaeus, Systema Naturae, p. 816 (original description, without illustration)

1830 *Carchesium polypinum*—Ehrenberg, Abh. dt. Akad. Wiss. Berl., Jahr 1830: 41 (combining author)

1838 *Carchesium polypinum* (Linne, 1758)—Ehrenberg, Infusionsthierchen, p. 278 (revision)

1854 *Carchesium polypinum*—Stein, Infusionsthiere auf ihre Entwickelungsgeschichte untersucht, p. 78 (redescription based on observation *in vivo*)

1922 *Carchesium corymbosum*—Penard, Etudes Infusoires, p. 260 (synonym; description with illustrations)

1935 *Carchesium (Vorticella) polypinum* Linne, 1758—Kahl, Tierwelt Dtl., 30: 738 (revision)

1962 *Carchesium polypinum* (Kent) Linne, 1758—Liebmann, Handbuch der Frischwasser und Abwasser-Biologie I, p. 363 (saprobiological characteristics)

1974 *Carchesium polypinum*—Foissner and Schiffmann, Protistologica, 10: 491, 504 (morphometric characterization and silverline system)

1992 *Carchesium polypinum* (Linnaeus, 1758) Ehrenberg, 1830—Foissner et al., Informationsberichte des Bayer. Landesamtes für Wasserwirtschaft, 5/92: 137–148, Figures 1–32 (revision)

*Carchesium polypinum* is a common freshwater species that has been reported many times (Linnaeus, 1758; Ehrenberg, 1830, 1838; Stein, 1854; Penard, 1922; Kahl, 1935; Liebmann, 1962; Foissner and Schiffmann, 1974; Foissner et al., 1992). An improved diagnosis based on previous and present data and a detailed redescription based on the Qingdao population are supplied.

#### Improved Diagnosis

The colony was up to 2,500 μm high. The stalk was dichotomously branched. The zooids were inverted bell-shaped, 35–140 × 35–70 μm *in vivo*. The peristomial lip was single-layered and moderately everted. One contractile vacuole was located at the ventral wall of the infundibulum below the level of the peristomial lip. The macronucleus was J-shaped. Infundibular polykinety 3 (P3) terminates adstomally at the same level as P1; the inner row of P3 was about half the length of the other two rows. There were transverse silverlines numbering 70–80 from the peristome to the trochal band and about 30–50 from the trochal band to the scopula. Freshwater is the habitat.

### Description Based on Qingdao Population

The zooids were usually inverted bell-shaped, 35–65 × 35–60 μm *in vivo* (Figures 10A,B, 11B–F). The peristomial lip was about 60–85 μm in diameter, single-layered, and moderately everted (Figures 10A,B, 11B,F). The peristomial disc was usually slightly elevated above the peristomial lip in fully extended zooids (Figures 10A,B, 11B,F). The pellicular striations were extremely fine (Figures 10C, 11E,M).

The cytoplasm was colorless, usually containing several gray or colorless granules. A single contractile vacuole was ventrally located below the level of the peristomial lip (Figures 10A,B, 11C). The macronucleus was J-shaped (Figures 10A,B, 11H,I). A micronucleus was not observed.

The colony was up to 2,500 μm tall. The stalk was dichotomously branched, and the spasmoneme was discontinuous, extending throughout colony (Figures 10E, 11A).

The oral ciliature was genus-typical. Haplokinety and polykinety make approximately 1.25 circuits around the peristome before entering the infundibulum (Figures 10D, 11J,H,I). The infundibular polykineties (P1–P3) had three rows (Figures 10D, 11J–K). The three rows of P1 were nearly equal in length (Figures 10D, 11J–K). P2 terminates adstomally at the convergence of P1 and P3 (Figures 10D, 11K). P2 converges adstomally with P1 (Figures 10D, 11K). The inner row of P3 was about half the length of the other two rows. P3 terminates adstomally at the same level as P1 (Figures 10D,K). Only one epistomial membrane was observed, located at the entrance of the infundibulum (Figures 10D, 11J). Germinal kinety lies parallel to haplokinety in the upper half of the infundibulum (Figures 10D, 11H). The trochal band consists of dikinetids, located about three-quarters of the way down the zooid length (Figures 11H,I).

The silverline system consists of closely spaced transverse silverlines, numbering about 75 (*N* = 3) between the peristome and the trochal band and about 40 (*N* = 3) between the trochal band and the scopula (Figures 10C, 11E,M).

### Molecular Data and Phylogenetic Analyses

The newly obtained SSU rDNA sequences of the five species have been deposited in the GenBank database with length (bp), GC content, and accession numbers as follows: *Zoothamnium arbuscula*—1,708 bp, 43.74%, MZ478144; *Zoothamnium hentscheli*—1,605 bp, 43.61%, MZ478143; *Zoothamnium weishanicum* n. sp.—1,570 bp, 43.31%, MZ478142; *Epicarchesium sinense* n. sp.—1,622 bp, 42.54%, MZ478145; and *Carchesium polypinum*—1,619 bp, 42.74%, MZ478146.

**FIGURE 13:**
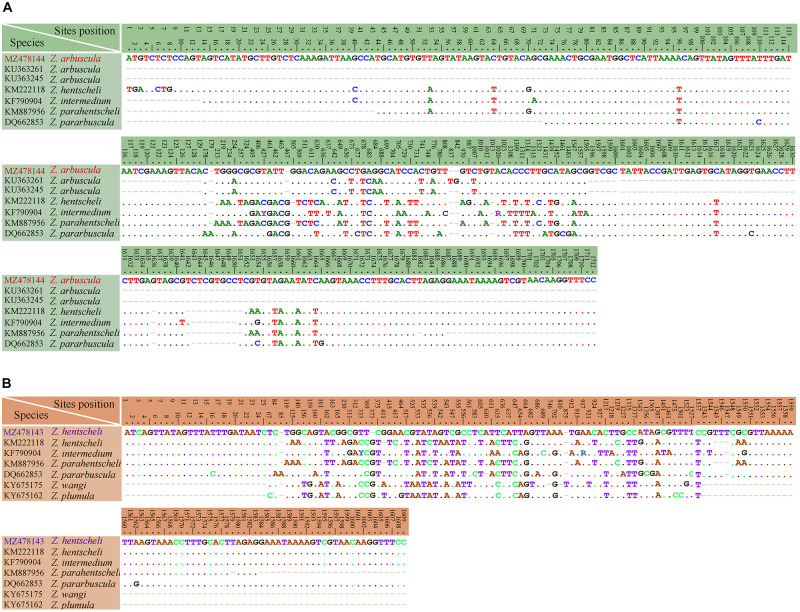
Table of nucleotide variations based on SSU rDNA sequences. The numbers in the header indicate the unmatched site positions. **(A)** *Zoothamnium arbuscula* (Weishan population) with other populations and similar congeners. **(B)** *Zoothamnium hentscheli* (Weishan population) with another population and similar congeners.

**FIGURE 14:**
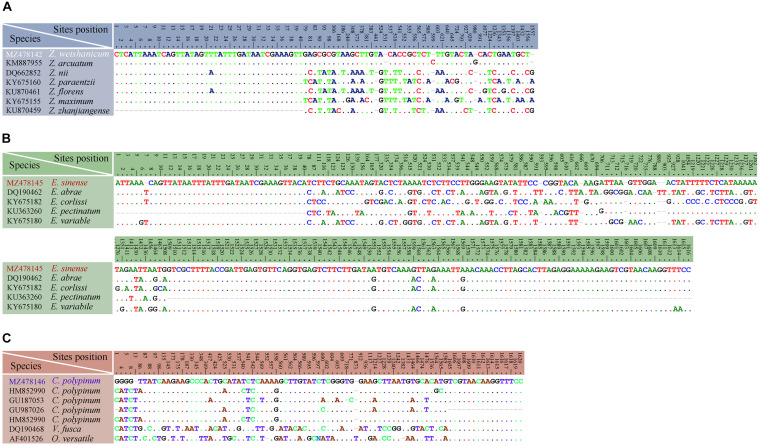
Table of nucleotide variations based on SSU rDNA sequences. The numbers in the header indicate the unmatched site positions. **(A)** *Zoothamnium weishanicum* n. sp. with similar congeners. **(B)** *Epicarchesium sinense* n. sp. with similar congeners. **(C)** *Carchesium polypinum* (Qingdao population) with other populations and similar congeners.

The phylogenetic trees based on SSU rDNA sequences using BI and ML methods have similar topologies; therefore, only the ML tree is shown here with support values from both algorithms (Figure 12). In the phylogenetic tree, the members of Zoothamniidae were grouped into three clades (clades I–III), resulting in the polyphyly of Zoothamniidae. Clade I clusters with one group of Epistylididae (ML 94% and BI 1.00), forming a clade that is sister to clade II (ML 97% and BI 1.00). Clade III is located outside the assemblage formed by clade I, clade II, and Epistylididae (ML 94% and BI 1.00). *Zoothamnium arbuscula* and *Z. hentscheli* nest within clade I. The new sequence of *Z. arbuscula* is sister to the previously reported sequence (KU363261), while the Weishan population of *Z. hentscheli* groups with *Z. arbuscula* rather than with *Z. hentscheli* (KM222118). *Zoothamnium weishanicum* n. sp. is located in clade II and is sister to *Z. arcuatum* with full support. *Epicarchesium sinense* n. sp. and *Carchesium polypinum* fall within the Vorticellidae assemblage. *Epicarchesium sinense* n. sp. clusters with *E. pectinatum* with moderate to high support (ML 81% and BI 1.00), forming a clade that is sister to the crown group comprising *Vorticella*, *Carchesium*, *Pseudovorticella*, and three species of *Epicarchesium* (*E. corlissi*, *E. variable*, and *E. abrae*). The Qingdao population of *C. polypinum* groups with two previously sequenced populations (GU187053 and HM852990) to form a clade that is sister to *Vorticella*. The molecular data based on the alignments of SSU rDNA sequences supports the validity of each of the five species investigated here and their separation from morphologically similar species (Figures 13, 14).

## Discussion

### Comments on *Zoothamnium arbuscula* Table 2

*Zoothamnium arbuscula* is a very common species that has been recorded many times (Ehrenberg, 1831, 1838; Ayrton, 1903; Wesenberg-Lund, 1925; Furssenko, 1929; Kahl, 1935; Biernacka, 1962; Müller, 1980; Xu, 1988; Foissner et al., 1992; Foissner and Berger, 1996; Shen and Gu, 2016). Ehrenberg (1831) gave the first description of this species and redescribed it 7 years later (Ehrenberg, 1838). Kahl (1935) made a revision including historical reports, a list of synonyms, an improved diagnosis, and notes on its distribution. He synonymized *Zoothamnium geniculatum* Ayrton, 1903 and *Z. geniculatum sensu* Wesenberg-Lund (1925) with *Z. arbuscula* and deemed that the marine population of *Z. arbuscula sensu* Kent (1880–1882) needed to be re-examined. Foissner et al. (1992) also made a revision of this species and questioned the identity of marine populations reported under the name *Z. arbuscula*. Ji et al. (2005a) and Wu et al. (2020) reported two marine species (*Z*. *pararbuscula* Ji et al., 2005 and *Z*. *apoarbuscula*, Wu et al., 2020, respectively) that are morphologically similar to *Z*. *arbuscula*. Thus, we speculate that *Z. arbuscula* is a freshwater species and the marine populations reported under the name *Z*. *arbuscula* are populations of *Z*. *pararbuscula*, *Z*. *apoarbuscula*, or other species.

**TABLE 2:** Comparison of *Zoothamnium arbuscula* (Weishan population) with other populations and closely related congeners.

Species	Zooid length *in vivo* (μm)	Zooid width *in vivo* (μm)	Colony height *in vivo* (μm)	Stalk	Spasmoneme surface	P2	Number of silverlines^a^	Habitat	Data source
*Z. arbuscula*	50–80	30–65	2,800–3,500	Narrowed at the basal part	Smooth	About twice as long as P3	46–52	FW	Present study
*Z. arbuscula*	40–70	–	Up to 6,000	Narrowed at the basal part	Smooth	–	–	FW	Ehrenberg, 1831; Kahl, 1935; Foissner et al., 1992
*Z. pararbuscula*	35–65	30–40	up to 1,500	Consistently evenly wide	Smooth	About twice as long as P3	25–35	MW	Ji et al., 2005a
*Z. apoarbuscula*	25–50	20–30	385–900	Narrowed at the basal part	Reticulated	About as long as P3	–	MW	Wu et al., 2020

*FW, freshwater; MW, marine water; P2, infundibular polykinety 2; P3, infundibular polykinety 3; –, data not available.*

*^*a*^from trochal band to scopula.*

Foissner et al. (1992) proposed the following diagnostic characteristics for the identification of *Z. arbuscula*: (i) differentiated zooids, microzooids that were bell-shaped, and macrozooids that were mostly ellipsoidal and rarely spherical; (ii) macronucleus that was usually C-shaped and located in the center of the zooid; (iii) a contractile vacuole was located at the dorsal wall of the infundibulum; (iv) the primary stalk was divided into three parts, including a basal part where the spasmoneme is absent and an upper part that is very thick; and (v) colony umbellate, each main branch feather-like. The Weishan population fits sufficiently well with all the above-mentioned characters and the original description of *Z. arbuscula* for us to conclude that they are conspecific.

*Zoothamnium arbuscula* is characterized by its umbellate colony shape and differentiated zooids, which distinguished it from most other congeners except *Z. pararbuscula* and *Z. apoarbuscula*.

*Zoothamnium pararbuscula* can be distinguished from *Z. arbuscula* by its shorter colony (1,500 vs. 2,800–3,500 μm *in vivo*), relatively smaller microzooids (35–65 × 30–40 vs. 50–80 × 30–65 μm *in vivo*), the even diameter of the primary stalk which is not conspicuously narrowed in the basal region (vs. uneven diameter and narrowed), and having fewer silverlines between the trochal band and the scopula (25–35 vs. 46–52) and its marine (vs. freshwater) habitat (Ji et al., 2005a).

*Zoothamnium apoarbuscula* differs from *Z. arbuscula* in having a shorter colony (385–900 vs. 2,800–3,500 μm tall *in vivo*), relatively smaller microzooids (25–50 × 20–30 vs. 50–80 × 30–65 μm *in vivo*), the reticulated surface of its spasmoneme (vs. smooth surface with granules) in the primary stalk, the P2 at about the same length as P3 (vs. P2 about twice as long as P3), and its marine (vs. freshwater) habitat (Wu et al., 2020).

### Comments on *Zoothamnium hentscheli* Table 3

*Zoothamnium hentscheli* was first described by Hentschel (1916) as “*Zoothamnium* sp. a.” and was renamed *Z*. *hentscheli* by Kahl (1935). Foissner et al. (1992) synonymized *Z. hentscheli* with *Z. kentii* Grenfell, 1884 based on both having a characteristic detritus coat. Wu et al. (2020) discussed the differences between them and considered that they are separate species, which is consistent with Ji et al. (2006, 2009).

**TABLE 3:** Comparison of *Zoothamnium hentscheli* (Weishan population) with other populations and closely related congeners.

Species	Zooid length *in vivo* (μm)	Zooid width *in vivo* (μm)	Branching pattern of stalk	Colony height *in vivo* (μm)	Number ciliary rows in P3	Habitat	Data source
*Z. hentscheli*	50–80	30–40	Alternate	Up to 1,500	3	FW	Present study
*Z. kentii*	90^a^	45^b^	Regularly dichotomous	Up to 2,300^c^	–	FW	Grenfell, 1884
*Z. kentii*	50–90	30–45	Irregular, usually alternate	Up to 2,300	–	FW	Foissner et al., 1992
*Z. hentscheli*	63–84	35–42	Irregular, usually alternate	Up to 1,200	–	FW	Hentschel, 1916; Kahl, 1935
*Z. parahentscheli*	50–75	30–40	Alternate	Up to 2,000	3	MW	Sun et al., 2009; Ji et al., 2015
*Z. apohentscheli*	40–65	25–40	Alternate	Up to 1,700	3	MW	Wu et al., 2020
*Z. wangi*	65–90	45–55	Alternate	Up to 1,000	2	MW	Ji et al., 2005b, 2011

*FW, freshwater; MW, marine water; P3, infundibular polykinety 3; –, data not available.*

*^*a*^1/285 inch in Grenfell (1884).*

*^*b*^Inferred from “the length being nearly twice the breadth” in Grenfell (1884).*

*^*c*^1/11 inch in Grenfell (1884).*

We identified the Weishan population as *Z. hentscheli* after comparing it with the original descriptions and redescriptions of all morphologically similar species of *Zoothamnium*. It closely resembles *Z. hentscheli* in the characteristic detritus coat on the zooids and stalk, the elongated inverted bell-shaped zooids, the single-layered and everted peristomial lip, the irregular alternately branched stalk, the shape and position of the macronucleus, the position of the contractile vacuole, and freshwater habitat. Thus, we identified our population as *Z. hentscheli*.

In addition to *Z. kentii*, one other species is very similar with *Z. hentscheli*, i.e., *Z. parahentscheli*, Sun et al., 2009, and two other congeners have a characteristic detritus coat, i.e., *Z. apohentscheli* Wu et al., 2020 and *Z. wangi* Ji et al., 2005. However, all of these are marine species, whereas *Z. hentscheli* is a freshwater species (Ji et al., 2005b, 2011, 2015; Sun et al., 2009; Wu et al., 2020). Furthermore, *Z. parahentscheli* can be easily separated from *Z. hentscheli* by its taller colony (up to 2,000 μm vs. up to 1,500 μm tall), wider primary stalk (20–28 vs.13–15 μm across), and shorter accessory branches (mostly 50–200 μm long vs. mostly over 300 μm long) (Sun et al., 2009; Ji et al., 2015). *Zoothamnium apohentscheli* differs from *Z. hentscheli* in having a smaller zooid (40–65 × 25–40 vs. 50–80 × 30–40 μm *in vivo*) (Wu et al., 2020). *Zoothamnium wangi* can be separated from *Z. hentscheli* by its smaller colony (up to 1,000 μm vs. up to 1,500 μm tall) and the two-rowed (vs. three-rowed) infundibular polykinety 3 (Ji et al., 2005b, 2011).

### Comments on *Zoothamnium weishanicum* n. sp. Table 4

*Zoothamnium weishanicum* n. sp. is characterized by its double-layered peristomial lip, slender zooids, alternately branched stalk, and freshwater habitat. Ji et al. (2005b) identified a population of *Z. duplicatum* sensu Kahl (1933) and an unnamed *Zoothamnium* population, i.e., *Zoothamnium* sp. sensu Kahl (1935) as populations of *Z. nii* Ji et al., 2005. Both populations were collected at Bremerhaven as epibionts of the hydrozoan *Cordylophora* sp. and identified as marine forms (Kahl, 1933, 1935). The original descriptions reveal that both populations closely resemble *Z. weishanicum* n. sp. in having a double-layered peristomial lip, an alternately branched stalk, a C-shaped macronucleus, and a dorsal contractile vacuole. Nevertheless, both differ from the latter in their marine (vs. freshwater) habitat. Furthermore, *Z. nii* can be separated from *Z. weishanicum* n. sp. by its wider zooids (40–50 vs. 30–45 μm *in vivo*), three-rowed (vs. two-rowed) infundibular polykinety 3, and marine (vs. freshwater) habitat (Ji et al., 2005b). Thus, we agree with Ji et al. (2005b) and accept that the two populations reported by Kahl (1933, 1935) are conspecific with *Z. nii*.

**TABLE 4:** Comparison of *Zoothamnium weishanicum* n. sp. with closely related congeners.

Species	Zooid length *in vivo* (μm)	Zooid width *in vivo* (μm)	Branching pattern of stalk	Number ciliary rows in P3	Habitat	Data source
*Z. weishanicum* n. sp.	55–90	30–45	Alternate	2	FW	Present study
*Z. arcuatum*	63–94	32–41	Irregular, usually alternate	3	BW	Ji et al., 2015
*Z. zhanjiangense*	80–100	45–55	Alternate	3	BW	Shen et al., 2017
*Z. nii*	70–80	40–50	Alternate	3	MW	Ji et al., 2005b

*BW, brackish water; FW, freshwater; MW, marine water; P3, infundibular polykinety 3.*

Only a few other species of *Zoothamnium* have a double-layered peristomal lip, two of which should be compared with *Z. weishanicum*, namely, *Z. arcuatum* Ji et al., 2015 and *Z. zhanjiangense* Shen et al., 2017. *Zoothamnium arcuatum* can be separated from *Z. weishanicum* n. sp. by the number of rows in infundibular polykinety 3 (three vs. two) and its brackish water (vs. freshwater) habitat (Ji et al., 2015). *Zoothamnium zhanjiangense* differs from *Z. weishanicum* n. sp. by its larger zooids (80–100 × 45–55 vs. 55–90 × 30–45 μm *in vivo*), three-rowed (vs. two-rowed) infundibular polykinety 3, and brackish water (vs. freshwater) habitat (Shen et al., 2017).

### Comments on *Epicarchesium sinense* n. sp. Table 5

Hitherto, only five species of *Epicarchesium* have been described: *E. abrae* (Precht, 1935) Ji et al., 2004, *E. corlissi* Sun et al., 2006, *E. granulatum* (Kellicott, 1887) Jankowski, 1985, *E. pectinatum* (Zacharias, 1897) Foissner et al., 1999, and *E. variable* (K sters, 1974) Sun et al., 2009.

**TABLE 5:** Comparison of *Epicarchesium sinense* n. sp. with congeners and morphologically similar species of *Carchesium*.

Species	Zooid length *in vivo* (μm)	Zooid width *in vivo* (μm)	Colony height *in vivo* (μm)	CV	Ma	PL	Pellicle with conspicuous cortical blisters	Stalk with septa	Main stalk with wedge-like structure	Pelagic	Habitat	Data source
*E. sinense* n. sp.	45–60	30–40	400–750	One, ventral	J-shaped	Single-layered	Yes	No	No	No	FW	Present study
*E. abrae*	45–85	35–55	300–500	One, dorsal	J-shaped	Single-layered	No	No	No	No	MW	Ji et al., 2004
*E. corlissi*	60–70	25–35	300	One, ventral	J-shaped	Double-layered	No	No	No	No	MW	Sun et al., 2006
*E. variabile*	100–120	50–65	400–500	One, ventral	J-shaped	Single-layered	No	No	No	No	MW	Sun et al., 2009
*E. pectinatum*	40–70	60	Up to 1,360	Two, ventral	J-shaped	Single-layered	Yes	No	Yes	Yes	FW	Foissner et al., 1999
*E. granulatum*	65–105	30–60	400	Two, ventral	C-shaped or 3-shaped	Single-layered	Yes	Yes	No	No	FW	Jankowski, 1985; Leitner and Foissner, 1997
*C. epistylis*	50	–	–	One, ventral	C-shaped	Single-layered	–	Yes	No	No	FW	Claparède and Lachmann, 1858; Kahl, 1935
*C. cyclopidarum*	50	30–35	300	One, ventral	C-shaped	Single-layered	–	No	No	No	FW	Nenninger, 1948; Stloukal and Matis, 1997

*CV, contractile vacuole; FW, freshwater; Ma, macronucleus; MW, marine water; PL, peristomial lip.*

*Epicarchesium abrae* can be distinguished from *E. sinense* n. sp. by the appearance of its pellicle (smooth or only slightly tuberculate vs. with conspicuous cortical blisters), the position of the contractile vacuole (dorsally located vs. ventrally located), the middle row of P3 terminating near the adstomal end of P2 (vs. terminating beyond the adstomal end of P1), and its marine (vs. freshwater) habitat (Ji et al., 2004).

*Epicarchesium corlissi* can be easily separated from *E. sinense* n. sp. by its more slender zooid shape (60–70 × 25–35 vs. 45–60 × 30–40 μm *in vivo*), double-layered (vs. single-layered) peristomal lip, shorter colony (300 vs. 400–750 μm tall), smooth (vs. tuberculate) pellicle, and its marine (vs. freshwater) habitat (Sun et al., 2006).

*Epicarchesium granulatum* can be distinguished from *E. sinense* n. sp. by having larger zooids (65–105 × 30–60 vs. 45–60 × 30–40 μm *in vivo*), two (vs. one) contractile vacuoles, and stalk with septa (vs. smooth stalk) (Jankowski, 1985; Leitner and Foissner, 1997).

*Epicarchesium pectinatum* can be easily separated from *E. sinense* n. sp. by its campaniform (vs. pyriform) zooids, two (vs. one) contractile vacuoles, taller colony (up to 1.36 vs. 400–750 μm tall), main stalk with (vs. without) wedge-like structures, and its pelagic (vs. sessile) lifestyle (Foissner et al., 1999).

*Epicarchesium variable* differs from *E. sinense* n. sp. by its larger zooid (100–120 × 50–65 vs. 45–60 × 30–40 μm *in vivo*), smooth (vs. tuberculate) pellicle, the adstomal end of P3 terminating above the adstomal end of P1 (vs. below the adstomal end of P1 in *E. sinense*), and its marine (vs. freshwater) habitat (Sun et al., 2009).

Considering that the pellicular striations of *Epicarchesium sinense* n. sp. *in vivo* are fine, it could easily be misidentified as a *Carchesium* species. To confirm that our new species is not a misidentified known *Carchesium* species, it should also be compared with two morphologically similar *Carchesium* species, i.e., *C. epistylis* Clapar de and Lachmann, 1858, and *C. cyclopidarum* Nenninger, 1948. *Carchesium epistylis* can be distinguished from *E. sinense* n. sp. by its C-shaped (vs. J-shaped) macronucleus and stalk with (vs. without) septa (Claparède and Lachmann, 1858; Kahl, 1935). *Carchesium cyclopidarum* can be easily separated from *E. sinense* n. sp. by its C-shaped (vs. J-shaped) macronucleus (Nenninger, 1948; Stloukal and Matis, 1997).

### Comments on *Carchesium polypinum* Table 6

*Carchesium polypinum* is a well-known peritrich with a global distribution and is widely used in studies of ecology, cytology, and genetics (Foissner et al., 1992; Miao et al., 2004; Gentekaki and Lynn, 2009, 2012; Boas et al., 2018; Vlaičević et al., 2021). However, many populations have been reported without morphological information or voucher specimens. Furthermore, the zooid shape and the size of *C. polypinum* collected from different environments were variable during our study. The Qingdao population matches closely the main characters of *C. polypinum* as described by Ehrenberg (1831, 1838) and as summarized in the revision by Foissner et al. (1992), including the following: (i) the single-layered and everted peristomial lip, (ii) the dichotomously branched stalk, (iii) the J-shaped macronucleus, (iv) the contractile vacuole located at the ventral wall of the infundibulum below the level of the peristomial lip, (v) the height and the shape of the colony, (vi) the pattern of the oral ciliature, and (vii) the freshwater habitat (Ehrenberg, 1830, 1838; Foissner et al., 1992). Because the zooids of the Qingdao population are smaller than those of other populations, we suggest that the size range of *C. polypinum* zooids should be extended.

**TABLE 6:** Comparison of *Carchesium polypinum* (Qingdao population) with other populations and closely related congener.

Species	Zooid length *in vivo* (μm)	Zooid width *in vivo* (μm)	Colony height *in vivo* (μm)	Ma	Stalk with septa	Number of silverlines from peristome to trochal band	Number of silverlines from trochal band to scopula	Data source
*C. polypinum*	35–65	35–60	2,400–2,500	J-shaped	No	71–77	36–47	Present study
*C. polypinum*	80–140	–	up to 2000	J-shaped	No	85–100	56–65	Ehrenberg, 1830; Kahl, 1935; Foissner et al., 1992
*C. epistylis*	50	–	–	C-shaped	Yes	–	–	Claparède and Lachmann, 1858; Kahl, 1935

*Ma, macronucleus; –, data not available.*

One other freshwater species with a similar zooid shape should be compared with the present population, namely, *C. epistylis* Claparede and Lachmann, 1850. *Carchesium epistylis* differs from *C. polypinum* by its C-shaped (vs. J-shaped) macronucleus and stalk with (vs. without) septa (Claparède and Lachmann, 1858; Kahl, 1935). Therefore, the identity of the Qingdao population as *C. polypinum* is not in doubt.

### Phylogenetic Analyses

The phylogenetic tree inferred from SSU rDNA sequence data shows that the genus *Zoothamnium* is non-monophyletic and the species are grouped into three clades, which is consistent with previous studies (Li et al., 2008; Zhuang et al., 2018; Lu et al., 2020; Wu et al., 2020). The Weishan population of *Zoothamnium hentscheli* and *Z. arbuscula* are nested within clade I along with *Z. arbuscula* (KU363261), *Z. pararbuscula*, and *Z. apoarbuscula*. Within this clade, however, *Z. hentscheli* does not cluster with *Z. hentscheli* (KM222118), although no morphological information is available for the latter, so its identity could not be confirmed. *Zoothamnium weishanicum* n. sp. clusters with *Z. arcuatum* with maximal support (100% ML and 1.00 BI) in clade II. These two sequences differ by only two base pairs and share several morphological similarities including zooid shape, the double-layered peristomial lip, the shape and the position of the macronucleus, and the position of the contractile vacuole. However, the oral ciliature differs significantly in that P3 is two-rowed in *Z. weishanicum* n. sp. but is three-rowed in *Z. arcuatum* (Ji et al., 2015).

As expected, *Epicarchesium* and *Carchesium* group within the family Vorticellidae. *Epicarchesium* is non-monophyletic, which is consistent with previous studies (Sun et al., 2016; Zhuang et al., 2018; Lu et al., 2020). *Epicarchesium sinense* n. sp. is most closely related to *E. pectinatum* which is supported by morphological and ecological data such as their pellicle with conspicuous cortical blisters and their freshwater habitat (Jankowski, 1985; Leitner and Foissner, 1997). The Qingdao population of *C. polypinum* groups with the other two populations of *C. polypinum* (GU187053 and HM852990). It is noteworthy that several of the sequences identified as *C. polypinum* differ significantly from each other. However, since most of these lack morphological information or voucher specimens, it is difficult to verify the species identity of these sequences.

## Data Availability Statement

The datasets presented in this study can be found in online repositories. The names of the repository/repositories and accession number(s) can be found in the article/supplementary material.

## Author Contributions

TW performed the experiments and drafted the manuscript. LD performed the phylogenetic section. ZW, HE-S, SA-F, YL, and AW checked all the data related and helped to improve the draft. BL and CW supervised and organized to complete the work. All authors read and approved the final manuscript.

## Conflict of Interest

The authors declare that the research was conducted in the absence of any commercial or financial relationships that could be construed as a potential conflict of interest.

## Publisher’s Note

All claims expressed in this article are solely those of the authors and do not necessarily represent those of their affiliated organizations, or those of the publisher, the editors and the reviewers. Any product that may be evaluated in this article, or claim that may be made by its manufacturer, is not guaranteed or endorsed by the publisher.

## Footnotes

1
http://guidance.tau.ac.il/ver2/

2
http://www.phylo.org
